# A novel method to address the association between received dose intensity and survival outcome: benefits of approaching treatment intensification at a more individualised level in a trial of the European Osteosarcoma Intergroup

**DOI:** 10.1007/s00280-019-03797-3

**Published:** 2019-03-16

**Authors:** Carlo Lancia, Jakob K. Anninga, Matthew R. Sydes, Cristian Spitoni, Jeremy Whelan, Pancras C. W. Hogendoorn, Hans Gelderblom, Marta Fiocco

**Affiliations:** 1 Mathematical Institute Leiden University, Niels Bohrweg 1, 2333 CA Leiden, The Netherlands; 2 Department Paediatric Oncology, Radboud University Medical Center, Geert Grooteplein Zuid 10, 6525 GA Nijmegen, The Netherlands; 3 MRC Clinical Trials Unit at UCL, Institute of Clinical Trials and Methodology, UCL and MRC London Hub for Trials Methodology Research, 90 High Holborn, London, WC1V 6LJ UK; 4 Mathematical Institute Utrecht University, Budapestlaan 6, 3584 CD Utrecht, The Netherlands; 5 Department of Epidemiology, University Medical Center Utrecht, Heidelberglaan 100, 3584 CX Utrecht, The Netherlands; 6 Department of Oncology, University College London Hospital, 235 Euston Rd, Fitzrovia, London, NW1 2BU UK; 7 Department of Pathology, Leiden University Medical Center, Albinusdreef 2, 2333 ZA Leiden, The Netherlands; 8 Department of Medical Oncology, Leiden University Medical Center, Albinusdreef 2, 2333 ZA Leiden, The Netherlands; 9 Department of Biomedical Data Science, Section Medical Statistics, Leiden University Medical Center, Albinusdreef 2, 2333 ZA Leiden, The Netherlands

**Keywords:** Bone tumour, Sarcoma, Chemotherapy, Personalised medicine

## Abstract

**Purpose:**

There is lack of consensus on the prognostic value of received high dose intensity in osteosarcoma survivorship. Many studies have not shown a clear survival benefit when dose intensity is increased. The aim of this study is to go beyond chemotherapy intensification by arm-wide escalation of intended dose and/or compression of treatment schedule, while conversely addressing the relationship between treatment intensity and survival at the patient level. The study focusses on the difference in outcome results, based on a novel, progressively more individualised approach to dose intensity.

**Methods:**

A retrospective analysis of data from MRC BO06/EORTC 80931 randomised controlled trial for treatment of osteosarcoma was conducted. Three types of post hoc patient groups are formed using the intended regimen: the individually achieved cumulative dose and time on treatment, and the increase of individual cumulative dose over time. Event-free survival is investigated and compared in these three stratifications.

**Results:**

The strata of intended regimen and achieved treatment yields equivalent results. Received cumulative dose over time produces groups with evident different survivorship characteristics. In particular, it highlights a group of patients with an estimated 3-year event-free survival much larger (more than 10%) than other patient groups. This group mostly contains patients randomised to an intensified regimen. In addition, adverse events reported by that group show the presence of increased preoperative myelotoxicity.

**Conclusions:**

The manuscript shows the benefits of analyzing studies by using longitudinal data, e.g. recorded per cycle. This has impact on the drafting of future trials by showing why such a level of detail is needed for both treatment and adverse event data. The novel method proposed, based on cumulative dose received over time, shows that longitudinal treatment data might be used to link survival outcome with drug metabolism. This is particularly valuable when pharmacogenetics data for metabolism of cytotoxic agents are not collected.

**Trial registration:**

ISRCTN86294690.

## Introduction

Received dose intensity (RDI) was first described by Hryniuk as the given dose (in mg/m^2^) during a certain time period [1]. This definition applies to either single cytostatic agents or drug combinations [2]. The prognostic value of high RDI in cancer survivorship has been discussed in several studies [3–8] and for many cancer types; however, this debate has never reached consensus [9, 10].

In osteosarcoma, RDI increase can be achieved by schedule compression supported by granulocyte colony-stimulating factor, e.g. by comparing 3-weekly doxorubicin plus cisplatin with a 2-weekly administration of the same agents at the same dosage [11]. This led to a 31% RDI increase and a significantly higher proportion of good histologic response in the intensified arm compared to the uncompressed control arm. Yet unexpectedly, an increase of RDI was not associated with a better survival, leading to the conclusion that histologic response was not a good surrogate marker tout court for survival outcome in osteosarcoma, because its prognostic value is limited to homogeneous groups of patients.

Nevertheless, treatment decisions have been often based on histologic response [12]. However, the recently closed EURAMOS-1 study showed no evidence that regimen intensification yields better survival outcome for poor histologic response [13]. Other studies have not shown a clear survival benefit when increasing dose intensity in osteosarcoma [14], perhaps indicating that the effect of treatment intensification should not be addressed by cohort comparison.

Treatment-induced toxicity has been shown to vary among different groups of age and gender [15]. Recently, it has been shown that variant expression of genes, involved in the metabolism of methotrexate, cisplatin and doxorubicin is related to progression-free survival in osteosarcoma [16, 17]. This finding suggests the presence of inter-patient heterogeneity for metabolism of cytotoxic agents. Finally, there is evidence that lymphopenia is an independent prognostic factor for overall and progression-free survival in several cancers [18].

Besides pharmacogenetics, there are a number of other factors driving differences in RDI, many of which vary at the patient level. The aim of this study is to gain insight into the treatment effects in osteosarcoma. Thus, we analyse the difference in outcome results that is observed by using a progressively more individualised approach to dose intensity.

## Patients and methods

### Patients

Data for this study were collected from the MRC BO06/EORTC 80931 (ISRCTN86294690) randomised controlled trial for patients with newly diagnosed, resectable high-grade osteosarcoma. The trial compared pathological response and survival outcome of the control regimen (Reg-C), six courses of 3-weekly doxorubicin (DOX, 75 mg/m^2^) and cisplatin (CDDP, 100 mg/m^2^), versus a dose-intense regimen (Reg-DI), same courses administered 2-weekly and supported by the hematopoietic growth factor G-CSF. Chemotherapy was given perioperatively. Surgical resection of primary osteosarcoma was scheduled at week 6 since the start of treatment, i.e. after 2 × DOX + CDDP for Reg-C and after 3 × DOX + CDDP for Reg-DI. Postoperative chemotherapy was intended to resume 3 weeks after surgery in both arms. The dataset consists of 497 consenting patients, prospectively enrolled between 1993 and 2002. More details about MRC BO06 can be found in the primary analysis of the trial [11].

### Sample selection

From the original sample, we excluded 19 patients who did not start chemotherapy (13) or reported an abnormal dosage of one or both agents (6, given dose > 1.25 × prescribed dose). The outcome of interest is event-free survival (EFS), defined as time from the end of therapy until the occurrence of the first event defined as: local recurrence, evidence of new or progressive metastatic disease, second malignancy, death, or a combination of these events. We set a landmark at 180 days since registration, since nearly all patients enrolled in MRC BO06 completed the allocated regimen. For the analysis, we further excluded 57 patients, who reported an event within day 180 since randomization (50) or had not completed therapy by day 180 since randomization (7).

### Calculating target, achieved and regulated RDI

Both target RDI (tRDI) and achieved RDI (aRDI) are obtained from the standardised cumulative dose *δ* and the standardised time on treatment *τ*. The quantities *δ* and *τ* are computed on the prescribed regimen for tRDI, and on individual patients’ treatment data for aRDI [19].

Regulated RDI (rRDI) extends the concept of aRDI by considering the longitudinal component: it is the evolution of the standardised cumulative dose *δ* over time. The new measure rRDI is a collection of values (*δ, τ*): each of these pairs corresponds to the cumulative dose and the cumulative time on treatment at the end of a completed cycle. More details about the computation of rRDI can be found in “Appendix 1”.

### Displaying of treatment data in the time–dose plane

Graphical techniques are used to visualise cumulative dose and treatment duration. We represent aRDI in the time–dose plane (*τδ*-plane), where the standardised time on treatment, *τ*, is on the *x*-axis and the standardised cumulative dose received, *δ*, on the *y*-axis. In the *τδ*-plane, we represent patients’ aRDI with points, while rRDI is represented by lines made of linear pieces. Each piece in the line corresponds to a cycle, where the slope represents the treatment intensity achieved in that period.

### Statistical methods

We formed strata of patients according to tRDI, aRDI, and rRDI, and estimated EFS by applying Kaplan–Meier methodology on each stratification. We performed a landmark analysis at 180 days since randomization to investigate the association between RDI and EFS in the following scenarios: (i) patients are divided on the allocated regimen ( or ) which leads to two strata of identical tRDI (, ); (ii) patients are divided into four strata based on similarity of aRDI (, , , ); (iii) patients are divided into four strata based on similarity of rRDI (, , , ).

For scenarios (ii) and (iii), we formed post hoc patient groups (strata) by applying *k*-means clustering (unsupervised machine learning) of aRDI and rRDI separately. The choice of defining four strata was motivated in previous research on aRDI [19]. The *k*-means clustering methodology provides homogeneous strata with respect to aRDI or rRDI. Patients who reported a similar cumulative dose in a similar time window (aRDI) are grouped together; patients who reported a similar course of treatment (rRDI) are grouped together. Details are provided in “Appendix 2”. For both aRDI and rRDI stratification, we also look at the distribution of tRDI across the strata and provide median values of *τ, δ*, and number of cycles.

All analyses are performed using Python 3.6.2 with pandas 0.20.2 [20], scikit-learn [21], and lifelines [22].

## Results

**Fig. 1:**
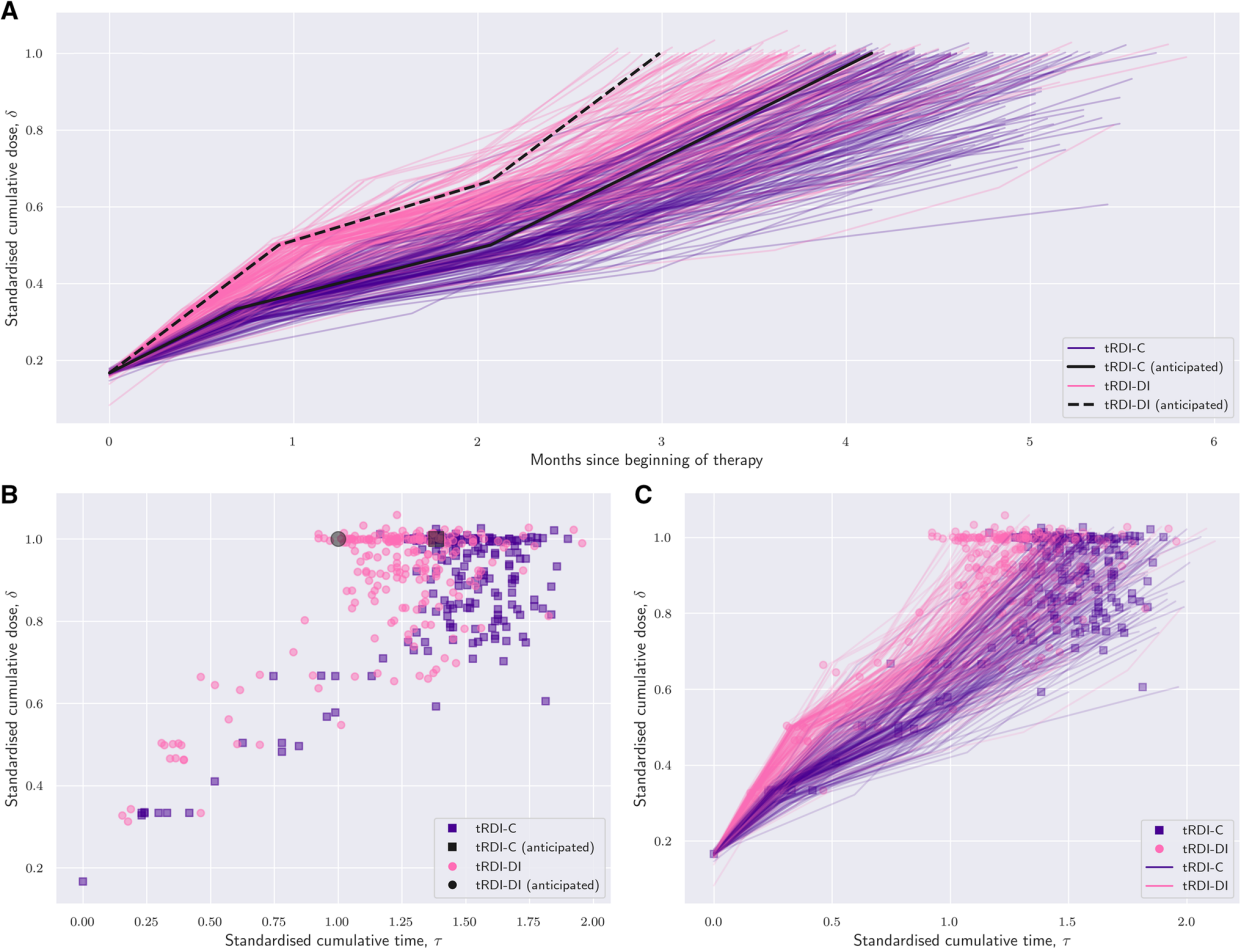
**a** Individual standardised dose over time coloured by the allocated regimen (pink: Reg-DI; purple: Reg-C); each line is a graphical representation of the regulated RDI (rRDI) of a patient; black lines show the anticipated (target) rRDI line of a patient randomised to Reg-DI (dotted) or Reg-C (solid); divergence of coloured lines from the target black one illustrates how complicated an individual course of treatment was. **b** Individual cumulative standardised dose vs standardised treatment duration; each point is a graphical representation of the achieved RDI of a patient (pink circle: Reg-DI; purple square: Reg-C); two thick black markers represent two fictitious patients who completed the protocol with no delays or dose reductions (dot: Reg-DI; square: Reg-C). These black markers are a graphical representation of target RDI. The larger the distance of a coloured point from the corresponding black one, the more complicated the individual course of treatment. With respect to **a**, this view does not show where the complications were located in time. **c** As end points of rRDI lines match the pattern of achieved RDI visualised in **b**, regulated RDI correctly extends achieved RDI over time

Figure 1 shows patients in the *τδ*-plane and the interrelationships between tRDI, aRDI, and rRDI. Figure 1a displays individual cumulative dose over time, i.e. rRDI lines, and compares them with those belonging to patients who reported no delays or reductions represented with the two thick black lines. Each line is obtained by joining the points (*τ, δ*) corresponding to each cycle. In Fig. 1b, aRDI and tRDI can be compared with patients who reported no delays or reductions, represented in the figure as black square (Reg-C target) and dot (Reg-DI target). Figure 1c shows that rRDI extends aRDI over time, because the pattern of the final rRDI-value is identical to that of Fig. 1b.

Each rRDI line presents a sharp change in the steepness in the central part of Fig. 1 top and bottom right: this marks the last preoperative cycle whose duration includes the surgery window. rRDI lines form a tight bundle in the early phase of the treatment, but later they open up in a hand-fan shape because treatment adjustments are generally more frequent towards the end of the protocol.

**Fig. 2:**
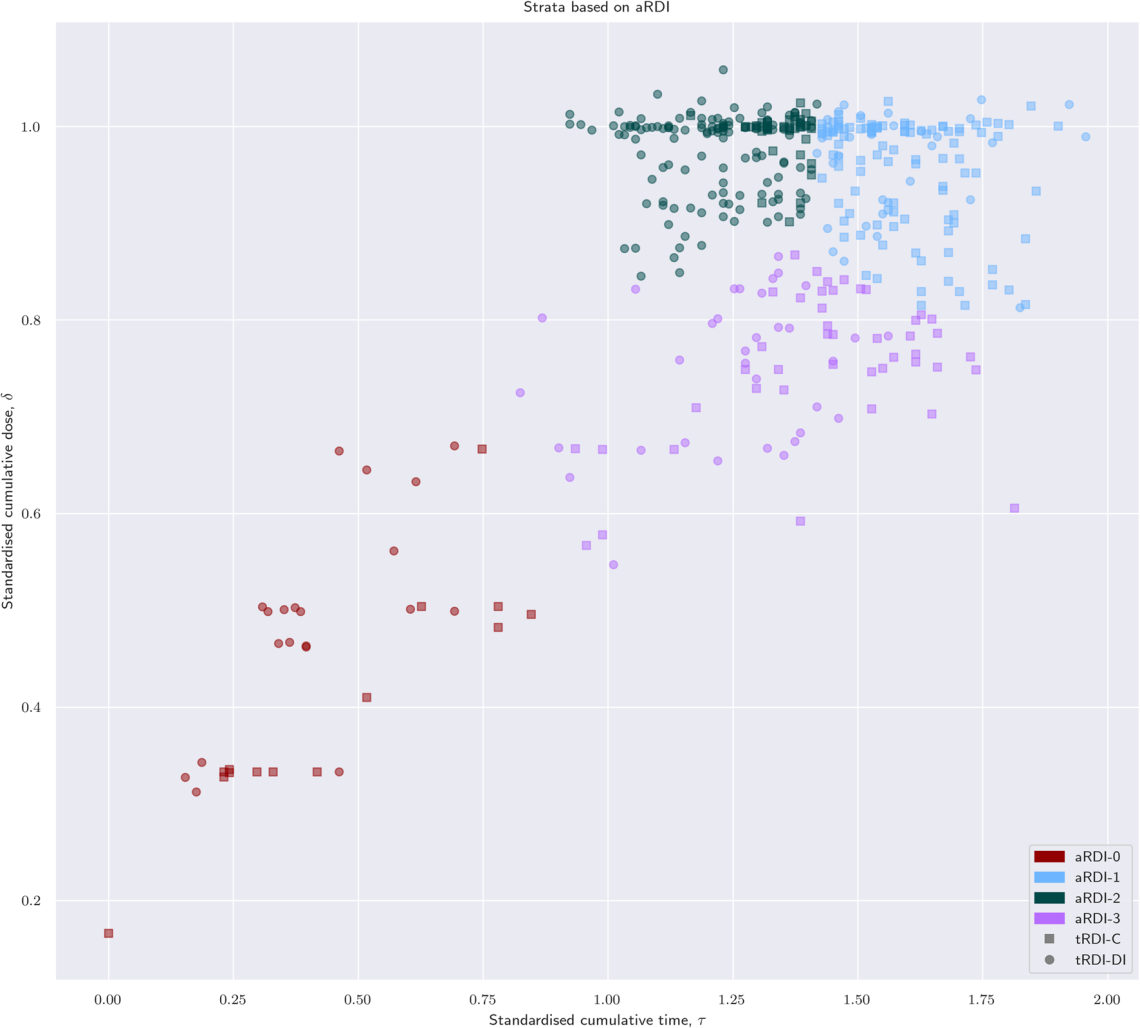
Individual cumulative standardised dose versus standardised treatment duration; patients are coloured in four groups determined by similarity of achieved RDI; patients are marked according to their target RDI value (circle: patient randomised to Reg-DI; square: Reg-C)

**Fig. 3:**
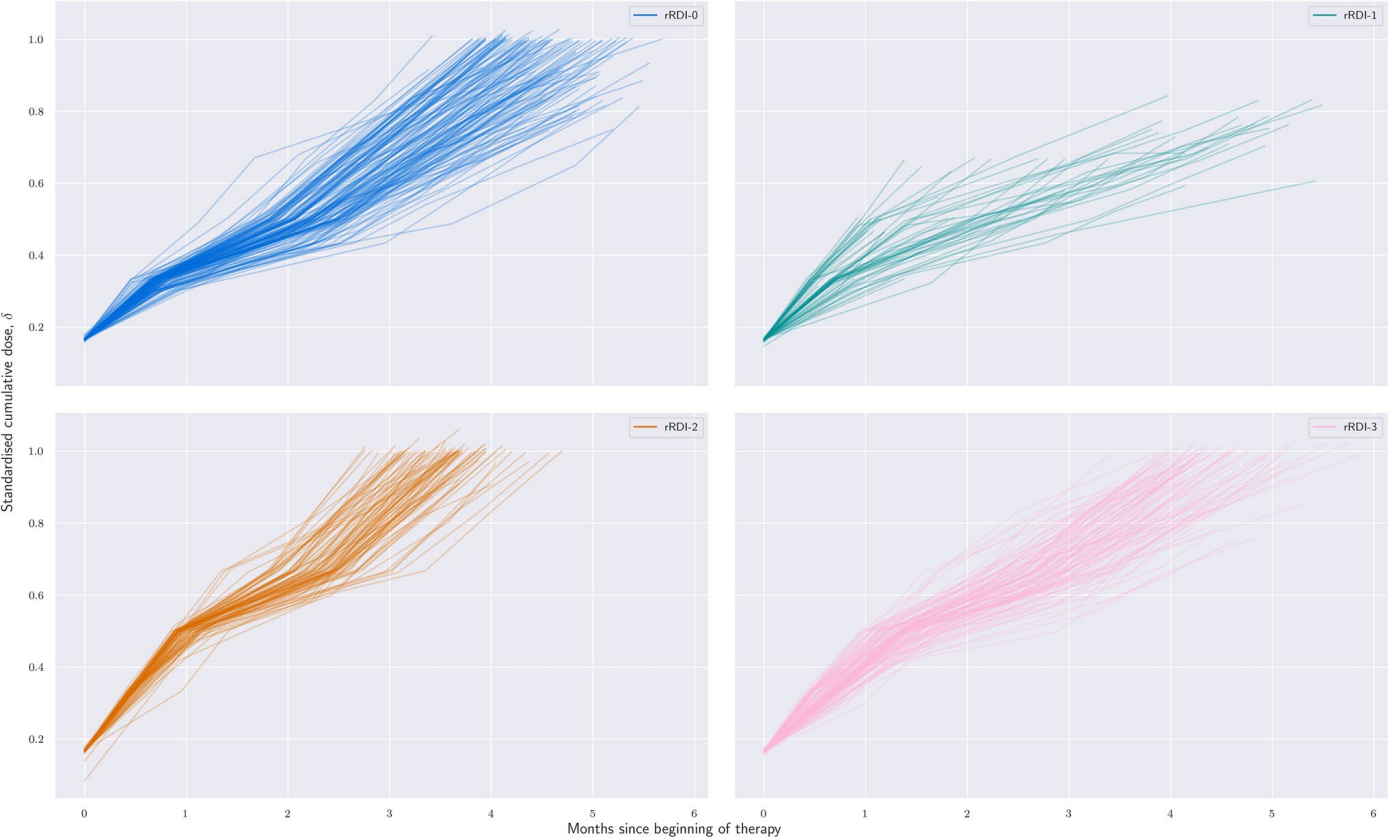
Grouping of patients by similarity of regulated RDI and individual rRDI lines of each group; each line shows the growth of the standardised cumulative dose received over time.  is mostly composed of patients randomised to Reg-C (92%),  captures treatment discontinuations and is balanced regimen-wise;  is composed only of patients allocated to Reg-DI;  is composed for the large majority (72%) by patients randomised to Reg-DI.  shows steeper lines than any other group, which means that patients therein reported the highest received dose intensity. In particular,  presents a marked difference with  in terms of line steepness that is more evident in the preoperative part of the regimen

Figure 2 shows the stratification based on aRDI; each colour represents a different post hoc strata. Strata are obtained at the landmark point—i.e. not at randomisation—by clustering patients with similar aRDI. Figure 3 shows rRDI-based stratification and individual rRDI lines for each group. Median values of relevant clinical quantities are reported in Table 1 for each group.

**Table 1:**
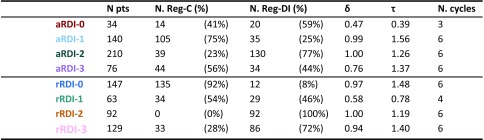
Characteristics of strata obtained by clustering patients on similar aRDI and rRDI

For each stratum, the table shows the total number of patients, percentage with respect to the allocated regimen, median values of standardised cumulative dose (*δ*), standardised cumulative time on treatment (*τ*), and number of cycles completed

The groups obtained by clustering of aRDI/rRDI can be interpreted as follows.

### aRDI strata (different colours correspond to different strata)

Stratum  is made of 34 patients of which 14 randomised to Reg-C (41%) and 20 to Reg-DI (59%). These are mostly patients who prematurely terminated the allocated protocol as the median cumulative dose received (*δ*) was 47% of the target one, while the median number of cycles completed is equal to 3.

Strata  is made of 140 patients, of which 105 were randomised to Reg-C (75%) and 35 to Reg-DI (25%). The median cumulative dose received was 99% of the anticipated one, with a median of six cycles completed, and the median duration of the treatment was 1.56 × 91 = 142 days. This is interpreted as the subgroup of Reg-C patients who had expected complications during their course of treatment, anticipated in the clinical trial protocol, and it also contains patients of the Reg-DI group who reported unexpected complications and required strong treatment adaptations (cf. Figs. 1b, 2).

Stratum  is made of 210 patients, of which 39 were randomised to Reg-C (23%) and 130 to Reg-DI (77%). The median cumulative dose is 100% of the target one, and the median duration of their allocated treatment was 1.26 × 91 = 115 days. Patients of the DI-group in this stratum completed their allocated treatment whilst reporting expected adaptations, while patients of the C-group completed their allocated treatment following quite closely the anticipated schedule. It can be interpreted as the subgroup of patients randomised to Reg-DI who had a course of treatment with expected complications, but it also contains patients allocated to Reg-C who reported little to no complications.

Stratum  is made of 78 patients with 44 randomised to Reg-C (56%) and 34 to Reg-DI (44%). The median cumulative dose is 76% of the anticipated one, the median number of completed cycles is six, and the median duration of the treatment is 1.37 × 91 = 125 days. As the cumulative dose in this group is always larger than 50% and the treatment duration is generally larger than 1 × 91 = 91 days (cf. Fig. 2), we conclude that this group is formed by a mixture of patients who terminated the allocated treatment in the postoperative phase or completed it reporting major reductions.

### rRDI strata (different colours correspond to different strata)

Stratum  is made of 147 patients, of which 135 were randomised to Reg-C (92%) and 12 to Reg-DI (8%). The median cumulative dose is 97% of the target one, and the median duration of their allocated treatment was 1.48 × 91 = 135 days. This group contains 67% of all C-patients.

Stratum  is composed of 63 patients, of which 34 were randomised to Reg-C (54%) and 29 to Reg-DI (46%). The median cumulative dose was 58% of the target one, the median number of cycles complete was four, and the median time on treatment was 0.78 × 91 = 71 days. These are patients who discontinued the protocol or experienced some complications during the course of treatment.

Strata  is made of 92 patients randomised to Reg-DI, which accounts for 42% of all patients randomised to this regimen; it does not contain patients from the C-group. The median cumulative dose was 100% of the target one, and the median duration of their allocated treatment was 1.19 × 91 = 108 days.

It is interesting to compare this group to : 129 patients, of which 33 were from the C-group (28%) and 86 from the DI-group (72%). The median cumulative dose was 94% of the target one, and the median duration of the protocol 1.40 × 91 = 127 days;  contains 39% of all DI-patients enrolled to the trial. Figure 3 shows that, compared to , the  group shows less steep rRDI lines, i.e. cycle-wise less intense treatments. Since most patients were randomised to the same regimen (Reg-DI), the treatment-intensity reduction is due to a higher rate of toxicity-induced treatment adaptations in ; see next section. Figure 3 also suggests that differences between  and  might be more evident in the preoperative phase.

### Adverse events in rRDI strata

**Fig. 4:**
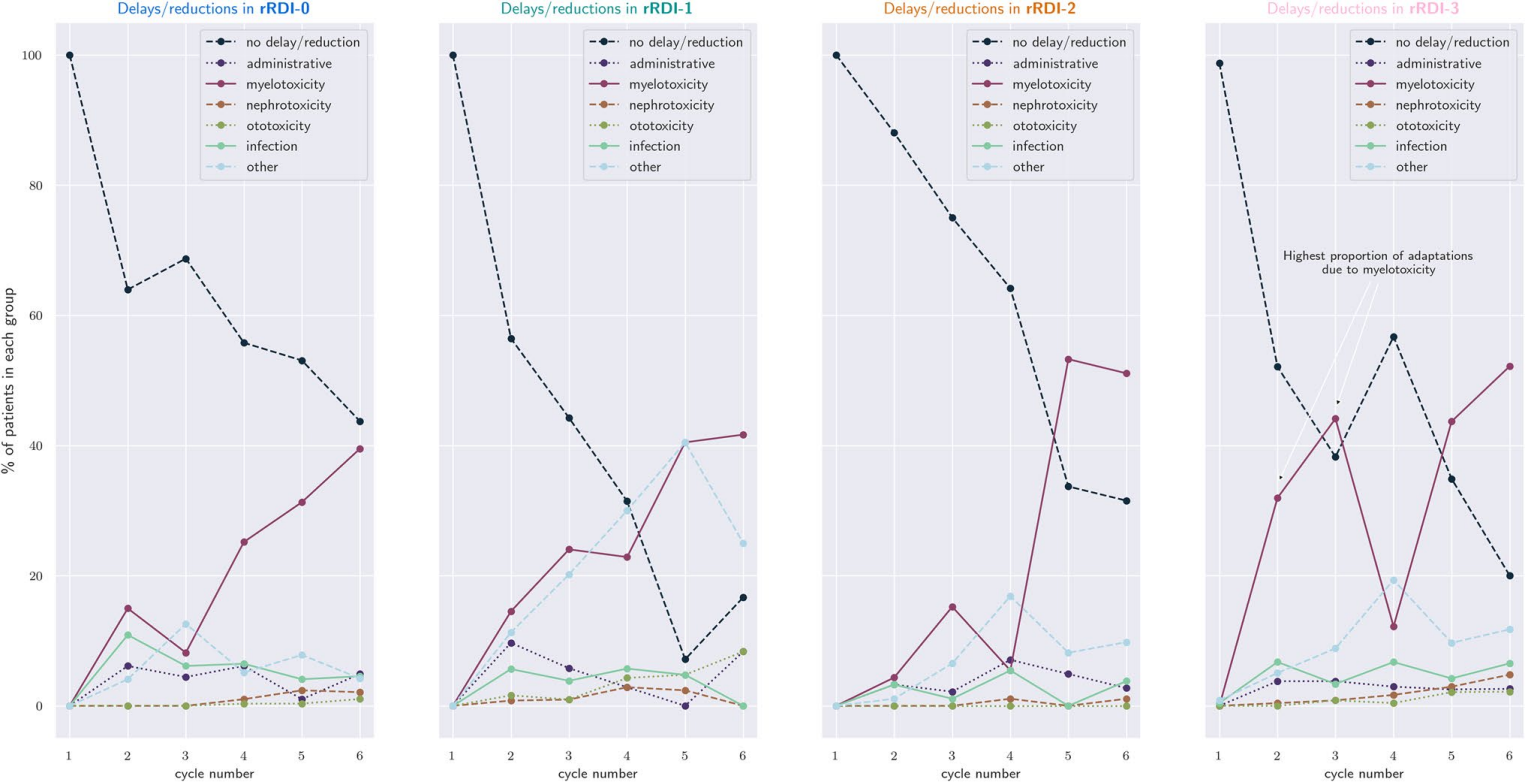
Proportion of patient cycles that required adaptations (either delays or dose reductions) according to the study protocol and corresponding cause of adaptation as reported by the case report form. The view is per rRDI-group, i.e. per group of patients with similar regulated RDI pattern. The *x*-axis displays cycle number, while the *y*-axis shows the proportion of patients in the group who required adaptations. Groups  and  are driven by late myelotoxicity, while groups  and  are driven by early myelotoxicity. In addition,  seems to be driven by ‘other’ reasons (most likely doxorubicin-induced cardiotoxicity, for which a dedicated checkbox was not present on the trial’s case report form), and  reports the highest proportion of adjustment due to myelotoxicity in the preoperative period. Cycles discontinued do not contribute to the plot

Figure 4 shows the proportion of patients in each rRDI stratum (relative to the group size) who required adaptations (either delays or dose reductions) according to the case report form. Each panel shows across cycles how many patients required treatment adaptations and the corresponding reason according to the case report form; cycles discontinued do not contribute to the plot. Groups  and  (especially the latter) are driven by late myelotoxicity, but groups  and  by early myelotoxicity. In addition,  seems to be driven by ‘other’ reasons (most likely doxorubicin-induced cardiotoxicity, for which a dedicated checkbox was not present on the trial’s case report form);  reports the highest proportion of adjustment due to myelotoxicity in the preoperative period.

### Survival in strata of tRDI, aRDI, and rRDI

**Fig. 5:**
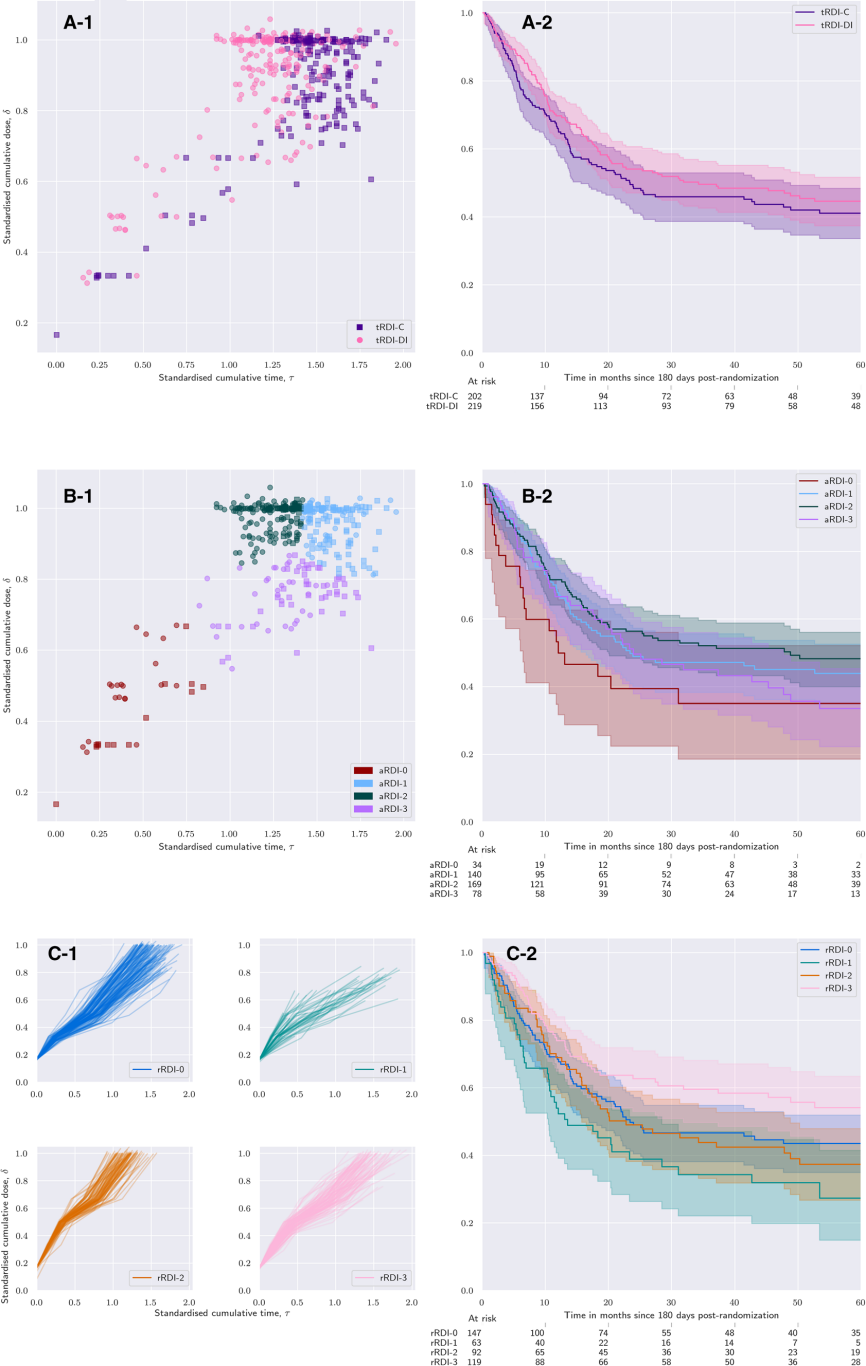
Estimation of event-free survival in groups that are homogeneous with respect to RDI using a progressively more individualised definition of RDI. **a-1** Groups defined with respect to target RDI and **a-2** the corresponding Kaplan–Meier curves; **b-1** groups defined with respect to achieved RDI and **b-2** the corresponding survival curves; **c-1** groups defined with respect to regulated RDI and **c-2** the corresponding survival curves

Figure 5 shows KM estimation of EFS obtained by stratifying patients according to tRDI (Fig. 5a-1, a-2), aRDI (Fig. 5b-1, b-2), or rRDI (Fig. 5c-1, c-2). For each estimated survival curve, the corresponding patient stratification is shown on the left panel.

EFS for strata based on tRDI (Fig. 5a-2) are similar to the survival based on strata of aRDI (Fig. 5b-2). KM curves for  and  (Fig. 5a-2) are very similar to those of  and  (Fig. 5a-2), respectively. This is to be expected as these strata largely overlap pairwise.

However, stratifying patients according to rRDI (Fig. 5c) offers a very different picture. Patients in the group  show an EFS very similar to patients from the  group (Fig. 5c-2). Strata  and  show largely overlapping survival curves, with 3-year EFS smaller than 50%. On the contrary,  (who had treatment-intensity reduction due to a high toxicity) shows better prognosis compared to strata  and , with 3-year EFS larger than 60%. Strata are based only on individual treatment data; thus, the method is unaware of the efficacy outcome.

## Conclusions

We presented a novel method to address the association between received dose intensity (RDI) and survival outcome by considering the treatment-intensity pattern achieved by each patient. This approach offers new insight into the direction of personalised treatment, extending beyond osteosarcoma.

We investigated event-free survival in patient groups that are homogeneous with respect to RDI at a progressively more individualised level. This showed the benefits of investigating the association between RDI and survival at the patient level rather than at the usual cohort level.

The application of the method was shown using data from MRC BO06, a randomised clinical trial in osteosarcoma. This is an interesting dataset because many studies in osteosarcoma have challenged the benefits of escalating the dosage and/or compressing the regimen cohort-wide, showing complex interrelationships between increased RDI, histologic response, and outcome [23, 24].

In this manuscript, we offered a novel tool for tackling the problem at the patient level. While grouping patients according to the target dose intensity overlooks inter-patient differences, clustering based on the achieved intensity levels at the end of treatment does not consider which treatment adaptation patterns led to individual reductions and delays during the treatment. Conversely, grouping patients on similarity of received dosage patterns takes into account also the individual tolerability to the regimen. As a result, we highlighted two subgroups of patients randomised to the dose-intense regimen who present quite different survival curves ( and ). Remarkably, the subgroup with the worst survival (, only patients from the DI-group and highest reported RDI) showed a KM curve that is nearly identical to the survival of the majority of patients from the C-group (). This finding seems to be linked to the individual tolerability because  and  (both mostly formed by patients of the DI-group) present differences in the individual treatment intensity reported, especially in the preoperative cycles (Fig. 3). A comparison with Fig. 7 from [14] suggests that the potential implications of this fact might be osteosarcoma specific.

We have also shown that longitudinal treatment data contain information about the link between adverse events and survival outcome (cf. Figs. 4, 5c). As such, longitudinal treatment data might be used to link survival with drug metabolism when pharmacogenetics data are unavailable, like for MRC BO06. This link has been already investigated in small cell lung cancer through average binding occupancies [25], a measure of which is a well-known predictor of both outcome and skin disorders. Studies in metastatic colorectal cancer showed the effectiveness and safety of therapy schemes adapted on adverse events [26] or targeting toxicity-related polymorphisms [27].

In conclusion, the strength of our method is the capability of detecting differences between patients randomised to the same regimen. Further, it seems to discriminate the prognostic value of chemotherapy-related complications over time. Indeed,  appears to be the subgroup of patients randomised to Reg-DI with the least tolerance to the regimen. In this respect, the manuscript offers a very precise view on the association between adverse events and osteosarcoma survival, which has been investigated through most severe chemotherapy-induced toxicity grades [28].

The method proposed here cannot be used yet in a prospective way. Yet, the results presented above suggest that there is room for gaining more insight into the effects of treatment intensification if longitudinal data and proper connected methodologies are used. On the one hand, our study calls for developing new methodologies targeted at cancer types where the link between treatment and survival is complex or confused. On the other, it calls for the collection of good-quality longitudinal data for both treatment and adverse events. Unfortunately, the level of detail available in the MRC BO06 dataset is not always accessible—an example is the recently concluded EURAMOS-1 trial. This in spite of treatment and adverse event data being longitudinal and already recorded per cycle in medical records. Clearly, making large datasets available with good-quality, longitudinal trial data might easily become an important issue for the organisation of future trials with respect to coordination efforts/overheads. This manuscript demonstrated that working in this direction might be a difficult, but rewarding approach.

## Appendix 1: Definition of tRDI, aRDI, and rRDI

Target received dose intensity (tRDI) is calculated as the ratio between standardised planned cumulative dose, *δ*, and standardised planned duration of regimen, *τ*. The former is obtained by standardising the sum of planned dose of all agents over all cycles of the allocated regimen; if a specific agent is not administered in a certain cycle, the contribution to the sum from that specific agent at that cycle is 0. Standardisation is performed by dividing the cumulative planned dose by the cumulative planned dose of a reference regimen. In MRC BO06, the cumulative planned dose is the same for each arm, 6 × (100 + 75) = 1050 mg/m^2^, therefore standardisation with respect to Reg-DI yields *δ* = 100% for both Reg-C and Reg-DI. The standardised planned duration of a regimen is obtained by standardising the difference in days between the planned start of the last and the first cycle of the regimen. Standardisation is performed by dividing the cumulative planned dose by the cumulative planned dose of a reference regimen. In MRC BO06, Reg-DI and Reg-C have planned duration of 91 and 126 days, respectively. Standardising with respect to Reg-DI yields a value of *τ* = 91/126 = 72%. The final computations of tRDI are as follows: for Reg-C, 1050/1050 × 91/126 = 72%; for Reg-DI, 1050/1050 × 91/91 = 100%.

Achieved received dose intensity (aRDI) is also calculated from standardised cumulative dose and standardised time-on-treatment, but these quantities are now computed on observed treatment data. For example, a patient reporting a 20% reduction in cycles 5 and 6 achieved a cumulative dose of 4 × (100 + 75) + 2 × (100 + 75) × 0.8 = 980 mg/m^2^, so *δ* = 980/1050 = 93%. In case an agent is omitted, the received dose of that agent in the formula is 0 for all the cycles affected by the omission; the same applies for all agents in case of discontinued cycles. Similarly, a patient who completed the protocol reporting a cumulative delay of 2 weeks (e.g. one 2-week delay or two 1-week delays at the beginning of two distinct cycles) would have *τ* = 105/91 = 115% if allocated to Reg-DI, or *τ* = 133/84 = 153% if allocated to Reg-C. In general, two patients—even two Reg-C or two Reg-DI—will report different values of *τ* and *δ* depending on the individual realisation of their intended treatment, i.e. depending on the delays and dose reductions reported throughout the treatment. In the graphical representation, the ratio *δ*/*τ* is the slope of a line connecting the patient point with the origin (0, 0) of the *τδ*-plane. For patients who completed all planned cycles, the quantity *δ*/*τ* is close to the aRDI as defined in [14]. For patients who did not complete all planned cycles, the ratio *δ*/*τ* might be difficult to use in practice. A solution is either to work with the pair (*δ, τ*) or to multiply *δ*/*τ* by the proportion of cycles completed.

Regulated received dose intensity (rRDI) is the evolution of the standardised cumulative dose over time; rRDI is a function of the time on treatment: for each time *t*, rRDI is the aRDI that patients would report in case of protocol discontinuation at exactly that moment. Both regimens of MRC BO06 were composed of six cycles, where both agents were administered at the beginning of each cycle. Thus, the standardised cumulative dose will increase at most six times during the administration of the regimen (it will increase less times only in case of discontinuation). This means that for MRC BO06, rRDI can be represented as a sequence of (at most) six values, i.e. the sequence of aRDI values that patients would report if they discontinued after each cycle was administered.

For a fictitious DI-patient, who completed the protocol without delays or dose reductions, rRDI can be displayed as the sequence of (*δ, τ*)-values [(0.167, 0/91), (0.333, 14/91), (0.500, 28/91), (0.667, 63/91), (0.833, 77/91), (1.000, 91/91)]; for the corresponding C-patient, [(0.167, 0/91), (0.333, 21/91), (0.500, 63/91), (0.667, 84/91), (0.833, 105/91), (1.000, 126/91)].

## Appendix 2: Clustering of patients based on achieved and regulated received dose intensity (aRDI and rRDI)

Clustering forms groups of patients such that members of the same group are similar with respect to received dose intensity.

Clustering based on achieved received dose intensity (aRDI) is performed by grouping patients with similar values of *δ* and *τ*. In other words, clustering returns groups of patients who were administered a similar cumulative dose in a similar time window. This means that patients assigned to the same cluster will report similar aRDI, or closer to aRDI of an average patient in the same group than to aRDI of average patient from other groups. In particular, this kind of clustering will not separate well Reg-DI and Reg-C patients. Instead, it will group Reg-DI patients with large cumulative delays (due to a problematic course of therapy) with Reg-C with a regular course of therapy; it will also tend to group Reg-DI patients with a regular course of therapy with Reg-C patients who reported less than average or no delays at all.

Clustering based on regulated received dose intensity (rRDI) is performed by grouping patients with a similar course of therapy. Groups are formed using the relative increase of the cumulative dose over time. As explained in “Appendix 1”, rRDI can be represented as a sequence of (*δ, τ*)-values, one pair for each cycle administered. In case of MRC BO06, there can be up to 6 (*δ, τ*)-pairs. In other words, rRDI of a MRC BO06 patients can be described by the sequence [(*δ*_1_, *τ*_1_), (*δ*_2_, *τ*_2_), …, (*δ*_6_, *τ*_6_)], where *δ*_*i*_ and *τ*_*i*_ are the standardised cumulative dose and time on treatment up to at the beginning of the *i*th cycle. The slopes are obtained as ρ_i_ = (*δ*_*i*+1_ − *δ*_*i*_)/(*τ*_*i*+1_ − *τ*_*i*_), *i* = 1, 2, …, 5. The slopes measure the intensity of the treatment between the *i*th and the (*i* + 1)th cycle: if no reduction was applied to the nominal dosage of cycle *i* + *1*, the increase (*δ*_*i*+1_ − *δ*_*i*_) equals 1/6 for both a Reg-DI and a Reg-C patient; if the start of cycle *i* + 1 was not delayed, the increase (*τ*_*i*+1_ − *τ*_*i*_) equals 14/84 for a Reg-DI patient and 21/84 for a Reg-C patient. As a consequence, *ρ*_*i*_ equals, respectively, 1 or 2/3 for a Reg-DI and a Reg-C patient who did not report delays or dose reductions in cycle *i* + 1. If a delay or a reduction is applied, then *ρ*_*i*_ is smaller. In case a patient completes less cycles than anticipated, some *ρ*_*i*_ cannot be calculated and are replaced by 0. Clustering is performed by grouping patients based on the similarity of the whole sequence of values [*ρ*_1,_*ρ*_2,_*ρ*_3,_*ρ*_4,_*ρ*_5_]. With this method, groups tend to separate better Reg-C and Reg-DI patients, because information on the intended treatment (duration of cycles) is embedded in *ρ*_*i*_ at the cycle level and not just regimen-wise. Moreover, *ρ*_*i*_ carries information about the medical interventions that occurred in each cycle, offering a surrogate measure of individual tolerability to the allocated regimen.

## Abbreviations

RDI: Received dose intensity
tRDI: Target RDI
aRDI: Achieved RDI
rRDI: Regulated RDI
EFS: Event-free survival
